# Urine/Plasma Neutrophil Gelatinase Associated Lipocalin Ratio Is a Sensitive and Specific Marker of Subclinical Acute Kidney Injury in Mice

**DOI:** 10.1371/journal.pone.0148043

**Published:** 2016-01-29

**Authors:** Tamás Kaucsár, Mária Godó, Csaba Révész, Miklós Kovács, Attila Mócsai, Norbert Kiss, Mihály Albert, Tibor Krenács, Gábor Szénási, Péter Hamar

**Affiliations:** 1 Institute of Pathophysiology, Semmelweis University, Budapest, Hungary; 2 MTA-SE “Lendület” Inflammation Physiology Research Group, Department of Physiology, Semmelweis University, Budapest, Hungary; 3 CEVA Phylaxia Ltd., Budapest, Hungary; 4 MTA-SE Tumor Progression Research Group, 1st Department of Pathology and Experimental Cancer Research, Semmelweis University, Budapest, Hungary; Emory University, UNITED STATES

## Abstract

**Background:**

Detection of acute kidney injury (AKI) is still a challenge if conventional markers of kidney function are within reference range. We studied the sensitivity and specificity of NGAL as an AKI marker at different degrees of renal ischemia.

**Methods:**

Male C57BL/6J mice were subjected to 10-, 20- or 30-min unilateral renal ischemia, to control operation or no operation, and AKI was evaluated 1 day later by histology, immunohistochemistry, BUN, creatinine, NGAL (plasma and urine) and renal NGAL mRNA expression.

**Results:**

A short (10-min) ischemia did not alter BUN or kidney histology, but elevated plasma and urinary NGAL level and renal NGAL mRNA expression although to a much smaller extent than longer ischemia. Surprisingly, control operation elevated plasma NGAL and renal NGAL mRNA expression to a similar extent as 10-min ischemia. Further, the ratio of urine to plasma NGAL was the best parameter to differentiate a 10-min ischemic injury from control operation, while it was similar in the non and control-operated groups.

**Conclusions:**

These results suggest that urinary NGAL excretion and especially ratio of urine to plasma NGAL are sensitive and specific markers of subclinical acute kidney injury in mice.

## Introduction

Acute kidney injury (AKI) is a common and serious problem in clinical nephrology. Twenty one percent of hospitalized adults experience AKI worldwide according to a recent meta-analysis [1]. The most frequent cause of AKI is ischemia-reperfusion injury of the kidney [2]. AKI is also increasingly recognized as a cause of chronic kidney disease [3]. Serious AKI can be easily detected by conventional markers of kidney function such as blood urea nitrogen or creatinine. Moreover, renal tubular functional test, like urinary specific gravity depend on the hydration status of the animal and are thus, difficult to standardize. Thus, in the absence of simple methods, a specific and sensitive marker of tubular injury is especially needed to detect mild AKI below the sensitivity of traditional kidney function markers.

Neutrophil gelatinase-associated lipocalin (NGAL) has been introduced as a biomarker of AKI [4], as NGAL is produced by injured tubular epithelial cells [5, 6]. Following its promising debut as an early biomarker for AKI after cardiac surgery [7] NGAL was frequently used to asses kidney damage in human subjects after kidney transplantation, contrast agent administration, or in the critically ill patients [4, 8, 9], and in mouse AKI models [4, 10, 11]. NGAL has 3 isoforms: most of the synthesized NGAL is monomeric (25 kDa) or dimeric (45 kDa) and only a small fraction is heterodimeric (135 kDa–complexed with gelatinase) [12]. Human renal tubular cells produce mainly the monomeric form and to some extent the heterodimeric form [12]. Unlike urea or creatinine retention, which are markers of the overall excretory function of the kidney, NGAL is considered to be a marker of acute tubular cell injury. Thus, NGAL may be more sensitive than urea [4] in ischemia-reperfusion or contrast induced (CI-) AKI [13].

Nevertheless, NGAL is not an exclusive kidney damage marker as plasma NGAL (pNGAL) can increase in patients due to epithelial injury, or neutrophil activation [14], however in the latter case, the dimeric form prevails [12]. Moreover, NGAL expression increased in humans as a consequence of intestinal [15] or bronchial [16] epithelial injury, inflammation or cancer [17]. The variety of NGAL sources can make it difficult to identify the underlying pathology. NGAL originating from the kidney after injury is largely excreted into the urine, and only a small fraction appears in the plasma [18]. Furthermore, NGAL from other organs is filtered in the glomeruli and is largely reabsorbed by proximal tubules if tubules are intact [19]. Thus, tubular injury results in a huge increase in urinary NGAL accompanied by a relatively small elevation in plasma NGAL, whereas other organ pathologies would elevate plasma but not urine NGAL.

Therefore, we hypothesized that urinary NGAL excretion or urine/plasma NGAL may be sensitive markers of subclinical AKI, which cannot be detected by conventional renal function markers. We measured NGAL in the plasma and urine, and blood urea retention after varying degrees of renal ischemia-reperfusion injury or non-operated and control-operated mice. Mild ischemia-reperfusion injury and control operation could be differentiated only with urinary/plasma NGAL but not with BUN. Neutrophil deficiency did not alter renal NGAL production, supporting the hypothesis that neutrophils are not a major source of NGAL in the mouse AKI model.

## Materials and Methods

### Animals

Twenty-week-old male C57BL/6 (Charles River, Germany) and bone marrow chimera (see below) mice weighing 30.3±4.8 g were maintained under standardized (light on 08:00–20:00 h; 40–70% relative humidity, 22 ± 1°C), specified pathogen-free (SPF) conditions, with free access to standard rodent chow (Altromin standard diet, Germany) and tap water. All procedures were performed in accordance with guidelines set by the National Institutes of Health (USA), the Hungarian law on animal care and protection, and was approved by the “Institutional Ethical Committee for Animal Care and Use” of Semmelweis University (registration numbers: XIV-I-001/2103-4/2012 and 22.1/321/3/2011).

The effect of neutrophil deficiency was tested with using bone marrow chimeras with a neutrophil-deficient Mcl-1^flox/flox^LysM^Cre/Cre^ hematopoietic system [20]. C57BL/6 recipients were irradiated with 11.5 Gy from a ^137^Cs source and were injected intravenously with unfractionated bone marrow cells from wild type (WT) or Mcl-1^flox/flox^LysM^Cre/Cre^ (referred to as Mcl-1^ΔMyelo^) mice on the C57BL/6 background [20, 21]. On average, the bone marrow cells from the femurs and tibias of one donor were injected into five recipients. Four weeks after transplantation, the circulating neutrophil numbers were determined by flow cytometry as described [20]. Neutrophils were defined as Ly6G-positive cells within a characteristic forward- and side-scatter gate.

### Kidney ischemia-reperfusion injury in mice

Unilateral renal ischemia-reperfusion (I/R) injury with contralateral nephrectomy was performed as described previously [22]. Briefly, the experiments were carried out using standard operating procedures. The intra-abdominal temperature was maintained using a heating pad (HK-3, DOPS, Czech Republic). The animals were narcotized by an intraperitoneal (ip.) injection of 80 mg kg^-1^ bw ketamine (CP-Pharma Handelsgesellschaft mbH, Burgdorf, Germany) and 4 mg kg^-1^ bw xylazine cocktail (CP-Pharma Handelsgesellschaft mbH, Burgdorf, Germany). Postoperative care included morphine hydrochloride (2.5 mg kg^-1^ bw sc after the operation) analgesia and ceftriaxone (Rocephin, 20 mg kg^-1^ once after operation, sc. Roche Hungary Ltd., Budaörs, Hungary) to prevent infectious complications. After decapsulation of the left kidney, the left renal pedicle was prepared and clamped to induce ischemia. Ischemia duration was based on our previous studies, which established 10-min ischemia as mild, 20-min ischemia as moderate and 30-min as severe ischemia with high mortality. The right kidney was removed in all cases to allow functional evaluation of the injured kidney. To clearly separate changes caused by renal ischemia from those caused by surgery and manipulation of the kidney, control mice were operated in the same manner but without renal pedicle clamping (control-operated, ctrl-op). An additional group of non-operated (non-op) mice was sacrificed without prior surgery for the evaluation of the marker changes caused by the invasive surgery performed in control-operated mice. The right kidney was used for control purposes. The effects of 10-, 20- and 30-min (n = 7-17/group) ischemia with 24 hours reperfusion were evaluated.

### Animal sacrifice, blood, urine and organ collection

The animals were sacrificed by cervical dislocation performed by trained personnel. Right before sacrifice, heparin (500 U/mouse; Merckle GmbH., Germany) was injected ip. and blood was collected from the thoracic cavity after cross-section of the vena cava. Blood was centrifuged at 1500 g for 8 min at 4°C to obtain plasma. The animals were perfused transcardially with 20 ml physiological saline pre-cooled to 4°C. The post-ischemic kidney was removed for further processing. Plasma and renal tissue samples were snap frozen in liquid nitrogen and kept at -80°C until use.

Urine was collected for 24 hours in metabolic cages (Techniplast, Italy). Urine samples were centrifuged at 3000 g for 20 min at 4°C to remove the sediment, and were stored at -20°C.

### Plasma urea and urinary creatinine measurements

Blood urea nitrogen (BUN) was measured from 32 μL total blood samples with Reflotron® Urea test stripes (Roche Diagnostics GmbH, Mannheim, Germany) on Reflotron® Plus device (Roche Diagnostics GmbH, Mannheim, Germany) as described in the manufacturer’s protocol.

Urine creatinine concentration was assessed with a colorimetric, enzymatic assay (Diagnosticum Ltd. Budapest, Hungary) in 96 well plates (Greiner Bio-One GmbH, Frickenhausen, Germany) according to the manufacturer’s instructions. Optical density was measured at 555 nm with the SpectraMax 340 Microplate Spectrophotometer (Molecular Devices, Sunnyvale, USA). Concentrations were calculated with SoftMax® Pro Software (Molecular Devices, Sunnyvale, CA).

### NGAL, IL-6 and p40 ELISA

The plasma and urinary NGAL levels were determined with a mouse Lipocalin-2/NGAL DuoSet ELISA Development kit (R&D Systems, Minneapolis, MI, USA), which according to the manufacturer detects the recombinant and homodimer mouse Lipocalin-2/NGAL. Detection of the heterodimer has not been tested. Plasma NGAL and IL-6 correlated in septic patients [23], and increased levels of IL-6 [24] and IL-12 [25] were also reported after surgery, in mice. Therefore, plasma IL-6, IL-12/IL-23 total p40 cytokine levels were measured with mouse IL-6 and IL-12/IL-23 total p40 ELISA Ready-SET-Go kits (eBioscience, San Diego, CA, USA), as described by the manufacturer. Shortly, 96 well plates (Nunc™ GmbH & Co. KG, Langenselbold, Germany, Denmark) were coated with the capture antibody, and the non-specific binding sites were blocked with reagent diluent (1% BSA in PBS, pH 7.2–7.4). Adequately diluted samples (10^3^ to 10^5^-fold for NGAL) were incubated in duplicates for 2 hours, and then the detection antibody was added. Next, Streptavidin-HRP was linked to the biotinylated detection antibody, followed by a short incubation with TMB Substrate (Sigma-Aldrich Chemie GmbH, Steinheim, Germany). A washing session (5 times with 300 μl of washing buffer) was performed after each step until the addition of the substrate solution. The enzymatic reaction was terminated by stop solution containing H_2_SO_4_. The optical density was measured with Victor3™ 1420 Multilabel Counter (PerkinElmer, Wallac Oy, Finland) at 450 nm with wavelength correction set to 544 nm. The NGAL concentrations were calculated with WorkOut software (Dazdaq Ltd., Brighton, England), using a four parameter logistic curve-fit.

### Estimation of filtered NGAL

To differentiate between hypoxic renal damage and other causes of elevated urinary NGAL excretion we calculated the quantity of filtered NGAL in non- and control-operated mice and in mice subjected to 10-min ischemia. First we estimated the GFR from the body weight based on previous observations on C57BL/6 mice (mean GFR: 951 ± 235 μL min-1 100 g-1 bw) [26] and calculated the volume filtered in 24 hours. As the mouse NGAL is around 21 kDa [27], it is freely filtered by the glomeruli [28]. Next, we calculated the amount of filtered NGAL according to the following equation:

$$\text{Filtered NGAL}={\text{P}}_{\left[\text{NGAL}\right]}\;*\;{\text{Vol}}_{\left[24\text{h GFR}\right]},$$

where P_[NGAL]_ is the plasma NGAL concentration and Vol_[24h GFR]_ is the 24-hour volume of the glomerular filtrate. In the severe (20-, 30- min) ischemia groups increased BUN indicated renal dysfunction, thus we could not calculate NGAL filtration in these groups.

### Histology and NGAL immunohistochemistry of renal tissue in tissue microarray (TMA) slides

Renal tissue samples fixed in 4% buffered formaldehyde were dehydrated and embedded in paraffin wax (FFPE) for histology and immunohistochemistry. The renal tissue injury was evaluated morphologically by tissue microarray (TMA) as described previously [29]. Briefly, blocks of 70-sample TMAs contained duplicates of 2 mm diameter cylinders cut by the computer-controlled puncher of the TMA Master Device (3DHISTECH Kft, Budapest, Hungary) from each FFPE kidney. For morphology and immunohistology, 4 μm thick sections were cut from the TMA blocks. Renal tubular necrosis, tubular dilation and cast formation were evaluated in Periodic acid-Schiff (PAS) stained TMA sections. Each tissue section was scored by a pathologist (MA) blinded to the origin of the tissue on a 0 to 4 scale as follows: 0 = no lesion; 1 = minimal or focal changes affecting less than 20% of the field; 2 = mild changes or the extension of the lesion to approx. 25% of the field; 3 = moderate changes or the extension of the lesion from 25% to 50% of the field; 4 = severe changes or the extension of the lesion to more than 50% of the field.

Renal tubular epithelial cell NGAL content was visualized by immunostaining. Dewaxed and rehydrated TMA sections were cooked in a 0.01M Tris-HCl and 0.1 EDTA buffer (TBS; pH 9.0) for 25 min at 100°C for antigen retrieval. The immunostaining involved consecutive incubations of TMA sections in 1% bovine serum albumin (BSA) in TBS (pH 7.4) for 15 min, rabbit anti-human NGAL IgG (1:100; R&D Systems, Minneapolis, MI, USA) for 16 h and in goat anti-rabbit IgG EnVision-peroxidase polymer kit (Dako, Glostrup, Denmark) for 40 min, all at room temperature. Tissue-bond peroxidase activity was developed with a DAB/H_2_O_2_ chromogen/substrate kit (Dako). Immunostained TMA slides were digitalized using a Pannoramic Scan instrument. Images were processed by Pannoramic Viewer (3DHISTECH). General staining intensity was quantified by ImageJ (NIH) software.

### RNA preparation

Total RNA was extracted from the upper third of the kidney with TRI Reagent® (Molecular Research Center, Inc., Cincinnati, OH, USA) according to the protocol provided by the manufacturer [30]. In brief, the frozen renal tissues were homogenized by an IKA® DI 18 basic grinder (IKA® Works do Brasil Ltda., Taquora, Brazil). Chloroform (Sigma-Aldrich, Inc., St Louis, MO, USA) was added to each sample and mixed by vortexing. The aqueous phase was separated from the organic phase by centrifugation. RNA was precipitated from the transferred aqueous phase with an equal quantity of isopropyl alcohol by incubation for 30 min at room temperature. The RNA pellet was washed twice with 75% ethyl alcohol, and dissolved in 100 μl RNase free water. The RNA pellet was treated with DNase I, RNase-free (Fermentas, St. Leon-Rot, Germany) to eliminate possible DNA contamination. The DNase was inactivated by phenol/chloroform extraction (Fluka, Sigma-Aldrich, Buchs, Switzerland). The RNA concentration and purity was assessed with a NanoDrop 2000c Spectrophotometer (Thermo Fisher Scientific, Wilmington, DE, USA). All RNA samples had an absorbance ratio (260 nm / 280 nm) above 1.8. To check RNA integrity, the samples were electrophoresed on 1% agarose gel (Invitrogen Ltd., Paisley, UK) in BioRad Wide mini-sub® cell GT system (Bio-Rad Laboratories, Inc., Hercules, CA, USA), and the ratio of 28S and 18S ribosomal RNA bands was calculated. The RNA solutions were kept at -80°C until further procedures.

### Quantitative real-time PCR analysis of NGAL mRNA expression in renal tissue

NGAL mRNA levels were measured by double-stranded DNA (dsDNA) dye based real-time PCR. Reverse transcription into cDNA was carried out by the High-Capacity cDNA Archive Kit (Applied Biosystem, Foster City, CA, USA) according to the manufacturer’s protocol. Briefly, 1 μg total renal RNA was reverse transcribed into cDNA with random hexamer primers at 37°C for 2 hours with Bio Rad iCycler™ Thermal Cycler (Bio-Rad Laboratories, Inc., Hercules, CA, USA). The NGAL (Lcn2) gene expression from kidney tissue homogenates was evaluated on the Bio-Rad C1000™ Thermal Cycler with CFX96™ Optics Module real-time PCR system (Bio-Rad Laboratories, Inc., Singapore). The PCR reaction was performed with Maxima™ SYBR Green qPCR Master Mix (Fermentas, St. Leon-Rot, Germany), according to the manufacturer’s protocol. Primers for NGAL were designed by the NCBI/Primer-BLAST online software (Fwd: ACg gAC TAC AAC CAg TTC gC; Rev: AAT gCA TTg gTC ggT ggg g) and synthesized by Integrated DNA Technologies (IDT, Inc., Coralville, IA, USA). The endogenous reference gene was GAPDH (Fwd: CCA gAA TgA ggA TCC CAg AA, Rev: ACC ACC TgA AAC ATg CAA CA). Primer annealing was set to 58°C and the melting curve was analysed to detect any abnormality of the PCR product. All samples were measured in duplicates and mRNA expressions were calculated using the relative quantification (ΔΔC_q_) method [31]. The efficiency of the qPCR reaction was also verified with standard curves.

### Statistical analysis

Results are presented as mean±standard error of the mean (SEM) unless otherwise indicated. Logarithmic transformation was performed if Bartlett’s test indicated inhomogeneity of variances. Continuous variables were compared using either one-way analysis of variance (ANOVA), followed by the Dunnett's multiple comparison post hoc test versus the control operated group, or two-way ANOVA with Tukey’s multiple comparisons test. Linear correlation was assessed with Pearson product-moment correlation coefficient. The null-hypothesis was rejected if the p value reached statistical significance (*: p<0.05, **: p<0.01, ***: p<0.001).

## Results

### Blood urea nitrogen (BUN) and renal histology in relation to the duration of renal ischemia

**Fig 1:**
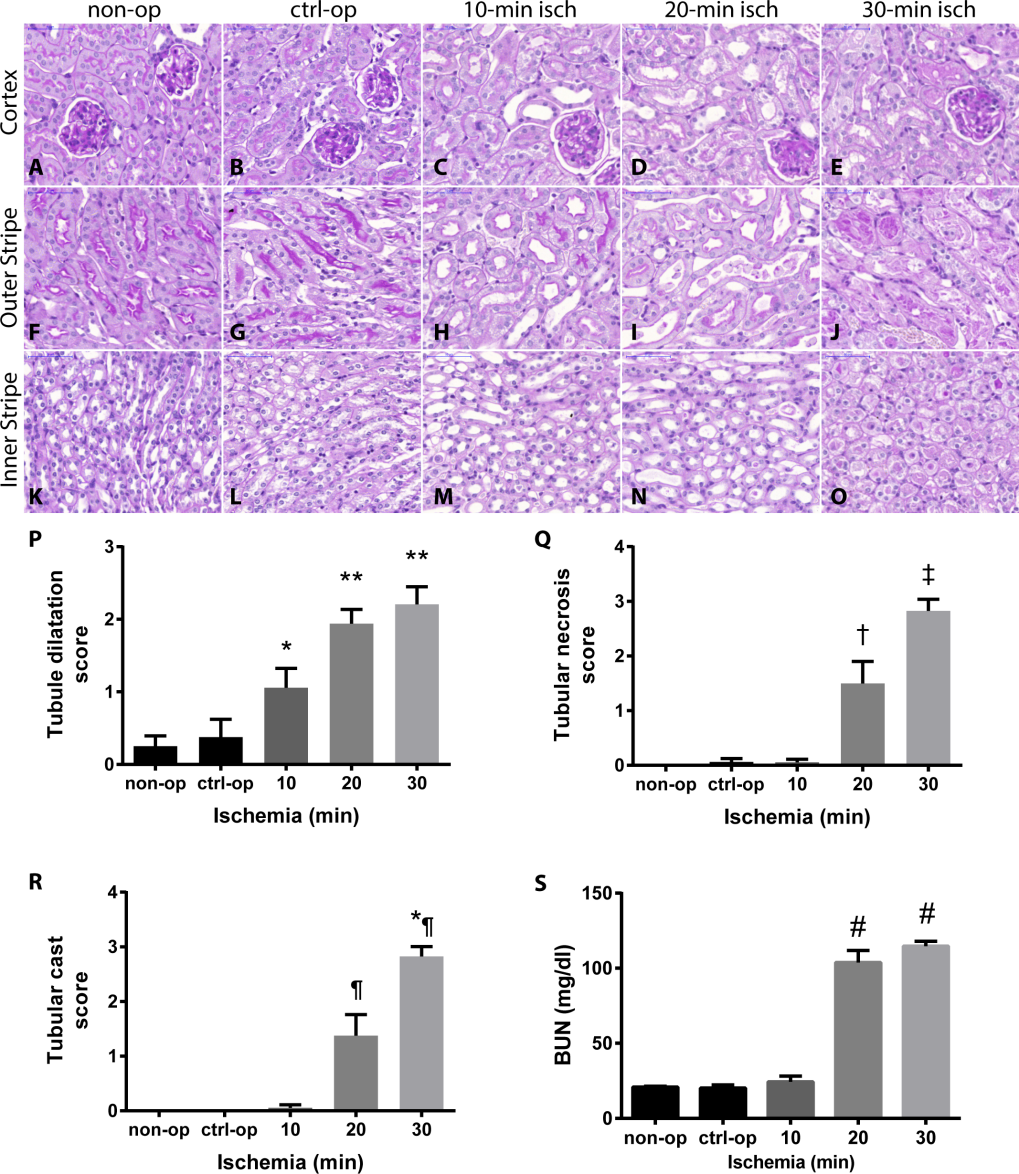
Severity of AKI after various renal ischemia times. The most sensitive parameter was (P) the tubular dilation score, which increased already after 10-min ischemia (C, H and M) compared to control-operated (ctrl-op; B, G and L) (* p<0.05 vs. non-operated (non-op) and p<0.001 vs. control-operated (ctrl-op); ** p<0.001 vs. non-op and ctrl-op). (Q) Tubular necrosis and (R) casts were present mostly after 20- (D, I and N) and 30-min (E, J and O) ischemia († p<0.05 vs. non-op, p<0.01 vs. ctrl-op, 10- and 30-min ishemia; ‡ p<0.0001 non-op, ctrl-op and 10-min ischemia, p<0.01 vs. 20-min ischemia; ¶ p<0.05 vs. non-op, p<0.01 vs. ctrl-op and 10-min ischemia, p<0.0001 vs. 30-min ischemia; *¶ p<0.0001 vs. non-op, ctrl-op, 10- and 20-min ischemia). There was no significant histologic change between non-op (A, F and K) and ctrl-op. (S) Renal function measured by blood urea nitrogen retention worsened after 20-min ischemia (# p<0.0001 vs. non-op, ctrl-op (ctrl-op) and 10-min ischemia). (non-op: n = 4; ctrl-op: n = 6; 10-min: n = 7; 20 min: n = 7; 30 min: n = 16).

BUN did not increase after 10-min renal ischemia (Fig 1S). Histological changes were also mild: only tubular dilation score was higher than in the control-operated group (Fig 1P), otherwise the kidneys had a normal histology in the 10-min renal ischemia group (Fig 1C, 1H, 1M, 1Q and 1R). After 20-min renal ischemia tubular necrosis and casts were obvious and quantified as elevated scores (Fig 1D, 1I, 1Q and 1R). Histological damage was also reflected functionally by higher BUN levels compared to control-operated mice in the 20-min renal ischemia group (Fig 1S). Thirty min renal ischemia caused extensive tubular necrosis with cast formation in the renal medulla (Fig 1J and 1O).

### Renal NGAL immunohistochemistry

**Fig 2:**
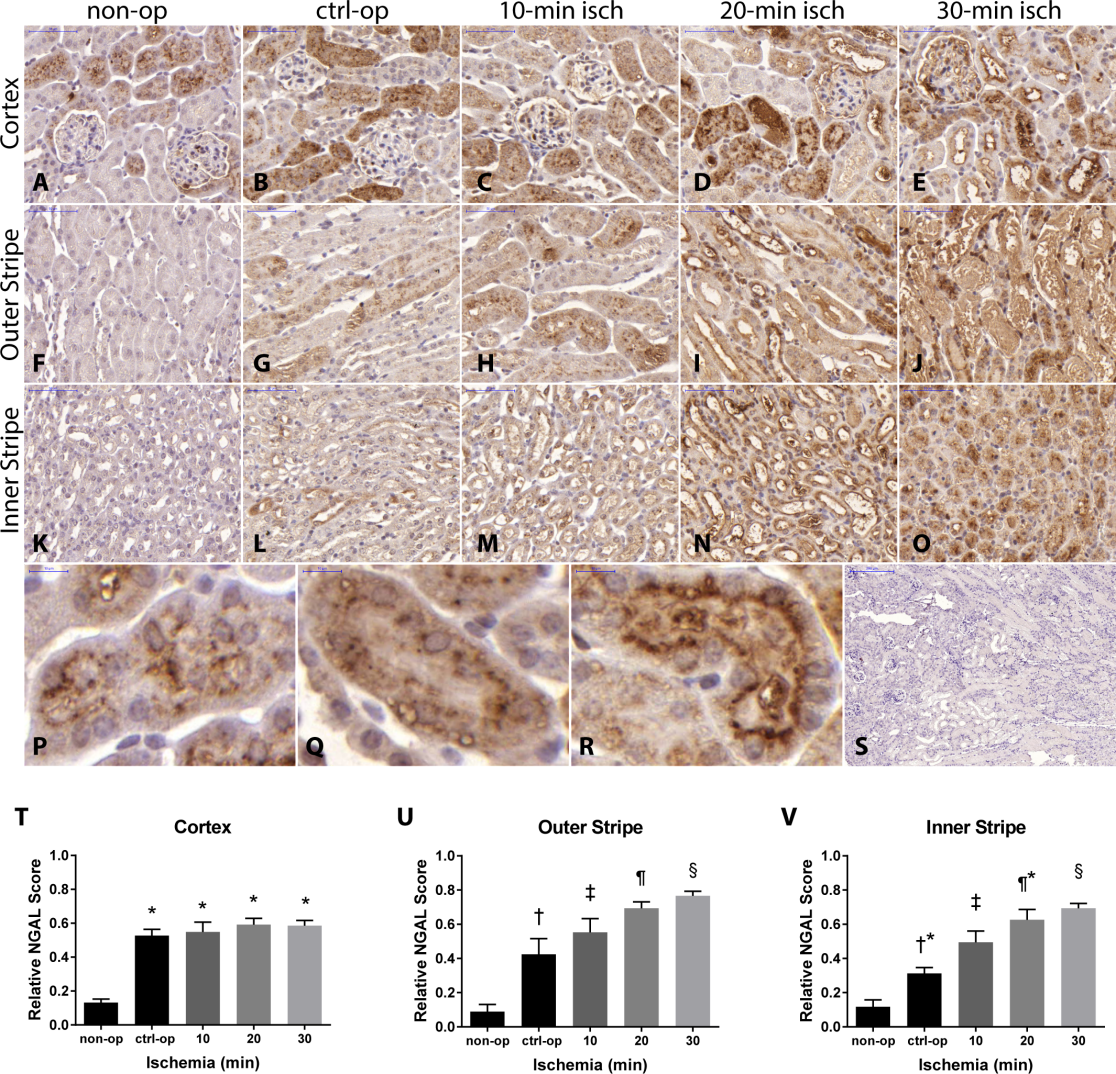
The intensity and extent of the NGAL immunostaining increased progressively with renal ischemia time. In the non-op kidneys (A, F and K) only the cortex (A) was NGAL positive, where the intracellular punctate staining pattern intensified after ischemia-reperfusion injury (R) compared to non-op (P) and control-op (Q). After control operation (ctrl-op; B, G and L) slight staining of the outer (G) and inner stripe (L) could be also observed. However, NGAL staining score increased further after 10- (C, H and M), 20- (D, I and N) and 30-min (E, J and O) renal ischemia in the outer medulla (U and V). Without the primary antibody no nonspecific staining was visible (S). * p<0.0001 vs. non-op; † p<0.05 vs. non-op and to 20-min, and p<0.001 vs. 30-min ischemia; ‡ p<0.001 vs. non-op, and p<0.05 vs. 30-min ischemia; ¶ p<0.0001 vs. non-op, and p<0.05 vs. ctrl-op; § p<0.0001 vs. non-op, p<0.001 compared ctrl-op, and p<0.05 vs. 10-min ischemia; †* p<0.01 vs. 20-min, and p<0.001 vs. 30-min ischemia; ¶* p<0.0001 vs. non-op, and p<0.01 vs. ctrl-op. (non-op: n = 4; ctrl-op: n = 7; 10 min: n = 9; 20 min: n = 8; 30 min: n = 17).

In non-operated mice a slight, diffuse cytoplasmic NGAL staining was present only in the renal cortex (Fig 2A). In control-operated mice, cortical NGAL staining area and intensity was larger, and NGAL also appeared in the medulla (Fig 2B, 2G and 2L). Ischemia-reperfusion injury increased NGAL staining further only in the medulla (Fig 2H–2J and 2M–2O). Interestingly, overall cortical NGAL staining area and intensity increased markedly but to a similar extent in all operated mice independent of the presence or absence of ischemia-reperfusion injury (Fig 2A–2E and 2T). On the other hand, increasing ischemia times were accompanied by a gradually increasing NGAL staining in the outer stripe of the medulla (Fig 2U). The area of NGAL staining was also proportional to the duration of ischemia in the inner stripe of the medulla (Fig 2V). Thus, only medullary but not cortical NGAL staining was influenced by ischemia time per se.

Besides an overall cytoplasmic staining we also observed a punctate NGAL staining pattern in tubular epithelial cells exclusively in proximal tubules (Fig 2P–2R). The punctate staining was present also in non- and control-operated mice (Fig 2P and 2Q) but it was more intense after severe (30-min) ischemia (Fig 2R). After severe (30-min) ischemia tubular casts and necrotic tubules were also NGAL positive (Fig 2J and 2O), while the control staining without the primary antibody proved to be negative (Fig 2S).

### Induction of NGAL after control operation and renal ischemia-reperfusion injury

**Fig 3:**
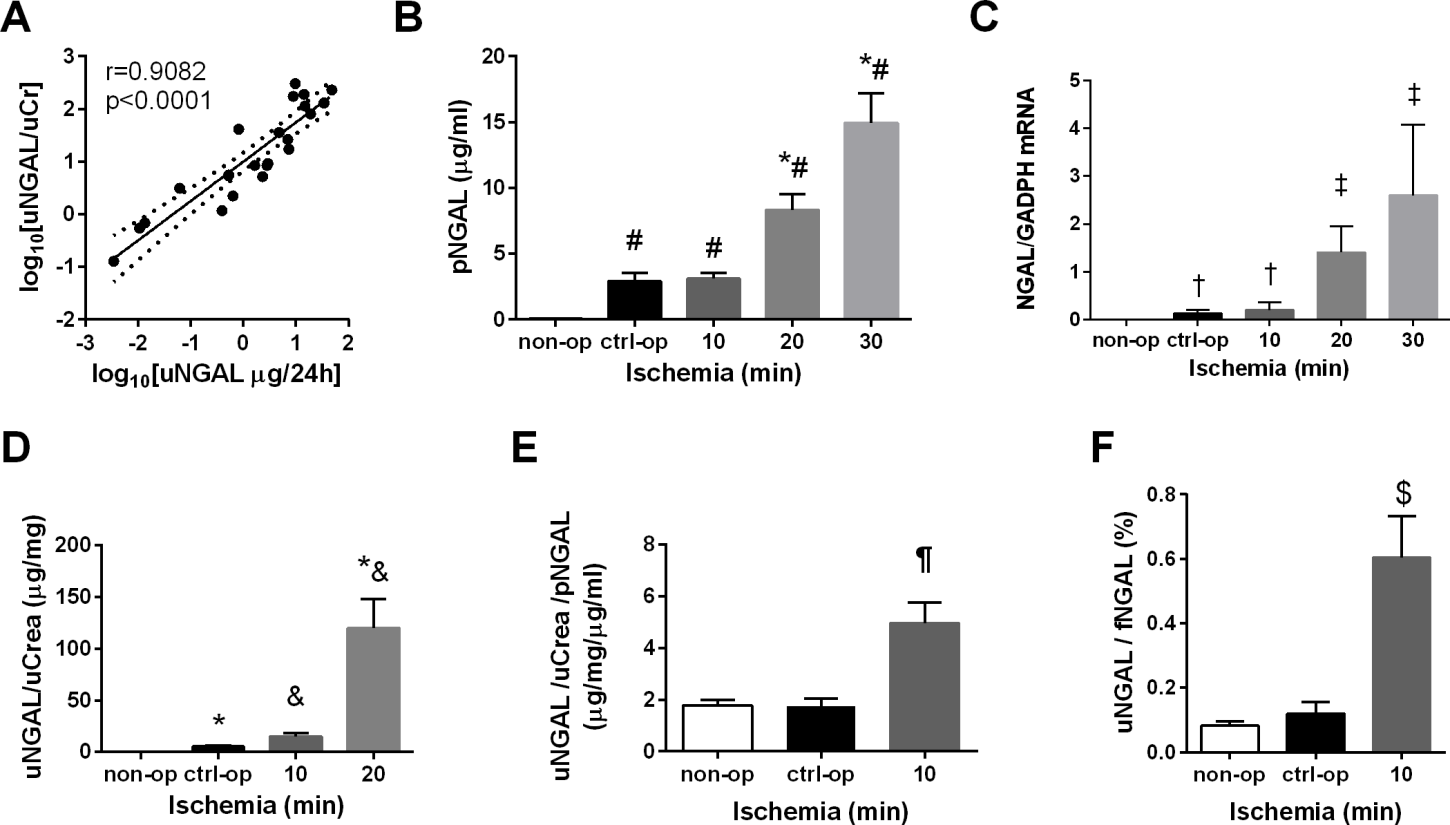
Urinary NGAL was sensitive enough to detect 10-min ischemia. (A) Urinary NGAL normalized to urinary creatinine correlated significantly with 24-hour urinary NGAL (r = 0.9082, p<0.0001). (B) Plasma NGAL levels and (C) NGAL renal mRNA expression increased both after 20-min ischemia and control operation (# p<0.01 vs. non-op, 20- and 30-min ischemia; *# p<0.01 vs. non-op and ctrl-op; † p<0.0001 vs. non-op, 20- and 30-min ischemia, ‡ p<0.0001 vs. non-op, ctrl-op and 10-min ischemia). (D) Urinary NGAL increased significantly after 10-min ischemia, but also after control operation (* p<0.0001 vs. non-op, p<0.05 vs. 10-min ischemia, p<0.0001 vs. 20-min ischemia; *& p<0.0001 vs. non-op and p<0.05 vs. ctrl-op; & p<0.0001 vs. non-op and ctrl-op). (E) Urinary NGAL further normalized to (divided by) plasma NGAL increased significantly after ischemia-reperfusion injury, but not after control operation (¶ p<0.001 vs. non-op and ctrl-op). (F) The ratio between urinary NGAL and calculated filtrered NGAL was significantly higher only after 10-min ischemia and not after ctrl-op vs. non-op ($ p<0.001 vs. ctrl-op and non-op). (non-op: urine and plasma n = 23, kidney RNA n = 10; ctrl-op: n = 6; 10 min: n = 6; 20 min: n = 5; 30 min: plasma and kidney RNA n = 17).

Normalization of urinary NGAL excretion to urinary creatinine gave similar results to that of 24-hour urinary NGAL excretion (Fig 3A). An intriguing finding of this study was that control operation significantly increased plasma NGAL concentration (Fig 3B), renal NGAL mRNA expression (Fig 3C), and urinary NGAL excretion (Fig 3D). Surprisingly, 10-min renal ischemia and control operation similarly increased renal NGAL mRNA expression (Fig 3C) and plasma NGAL concentration (Fig 3B). However, urinary NGAL excretion increased much more after 10-min renal ischemia than after control operation (Fig 3D). Thus, only urinary NGAL excretion differentiated 10-min renal ischemia from control operation. Twenty-, 30-min ischemia resulted in drastic elevations of renal NGAL mRNA expression (Fig 3C) and plasma NGAL concentration (Fig 3B). Urinary NGAL excretion markedly increased after 20-min ischemia in comparison to 10-min ischemia (Fig 3D).

### Urinary NGAL excretion to plasma NGAL level ratio (u/pNGAL)

We calculated the ratio of urinary NGAL excretion to plasma NGAL levels. Importantly, u/pNGAL values were similar in the control-operated and non-operated groups (Fig 3E). However, (u/pNGAL) significantly increased after 10-min ischemia compared to the control-operated mice, and was further elevated after more severe ischemia (Fig 3E). In order to better understand the difference in urinary NGAL excretion after 10-min ischemia and control operation we compared the excreted urinary NGAL to the estimated filtered NGAL in both groups. The excreted urinary NGAL was about 6-fold higher than the filtered NGAL after 10-min ischemia while they were similar after control and no operation (Fig 3F), suggesting that the post-ischemic kidney added a large amount of NGAL to the excreted fraction.

The diagnostic value of u/pNGAL in comparison to other kidney injury markers was further evaluated using Receiver Operating Characteristics (ROC) curve analysis. Only urinary NGAL discriminated successfully 10-min ischemia from control operation (Table 1). Furthermore, only u/pNGAL values discriminated 10-min ischemia from control operation, when tested against the non-operated controls (Table 2). On the other hand, plasma NGAL levels and renal expression of NGAL mRNA discriminated only the more severe (20-min and 30-min) ischemia from control operation (Table 1). Plasma NGAL levels or renal mRNA expression of NGAL also discriminated control-operated from non-operated (Table 2).

**Table 1:** The ability of plasma and urinary NGAL and BUN levels to discriminate between the severity of renal ischemia-reperfusion injury compared to control operation.

Ischemia vs. control-op.	10 min				20 min			
Kidney damage marker	Area	SE	P	Cut-off	Area	SE	P	Cut-off
BUN (mg dl-1)	0.60	0.16	0.52	N/A	1.00	0.00	**0.01**	46.920
NGAL mRNA	0.67	0.16	0.32	N/A	1.00	0.00	**0.01**	0.410
pNGAL (μg ml-1)	0.58	0.18	0.63	N/A	0.93	0.08	**0.05**	3.498
uNGAL (μg 24h-1)	0.86	0.14	**0.01**	2.385	0.90	0.10	**0.05**	0.733
uNGAL/uCrea (μg ml-1)	0.94	0.07	**0.05**	6.655	1.00	0.00	**0.01**	24.830
uNGAL/uCrea/pNGAL (μg mgCrea-1 μg-1 ml-1)	0.97	0.04	**0.01**	2.335	1.00	0.00	**0.01**	3.879

The area under the receiver operating characteristic (ROC) curve (AUC), standard error (SE) of the AUC, the P value (vs. control-op) and the cut-off value with the highest sensitivity and specificity are presented.

**Table 2:** The ability of plasma and urinary NGAL and BUN levels to discriminate between 10-min renal ischemia and control operation compared to non-operated (non-op.) group.

Operation vs. non-op.	Control-operated				10-min ischemia			
Kidney damage marker	Area	SE	P	Cut-off	Area	SE	P	Cut-off
BUN (mg dl-1)	0.58	0.15	0.51	N/A	0.54	0.15	0.76	N/A
NGAL mRNA	1.00	0.00	**0.01**	0.030	1.00	0.00	**0.001**	0.034
pNGAL (μg ml-1)	1.00	0.00	**0.001**	0.604	1.00	0.00	**0.001**	1.118
uNGAL (μg 24h-1)	1.00	0.00	**0.001**	0.250	1.00	0.00	**0.001**	1.271
uNGAL/uCrea (μg ml-1)	1.00	0.00	**0.001**	0.775	1.00	0.00	**0.001**	4.066
uNGAL/uCrea/pNGAL (μg mgCrea-1 μg-1 ml-1)	0.53	0.10	0.81	N/A	0.88	0.06	**0.01**	2.474

The area under the receiver operating characteristic (ROC) curve (AUC), standard error (SE) of the AUC, the P value (vs. non-op) and the cut-off value with the highest sensitivity and specificity are presented.

### Inflammation after 20- and 30-min renal ischemia

**Fig 4:**
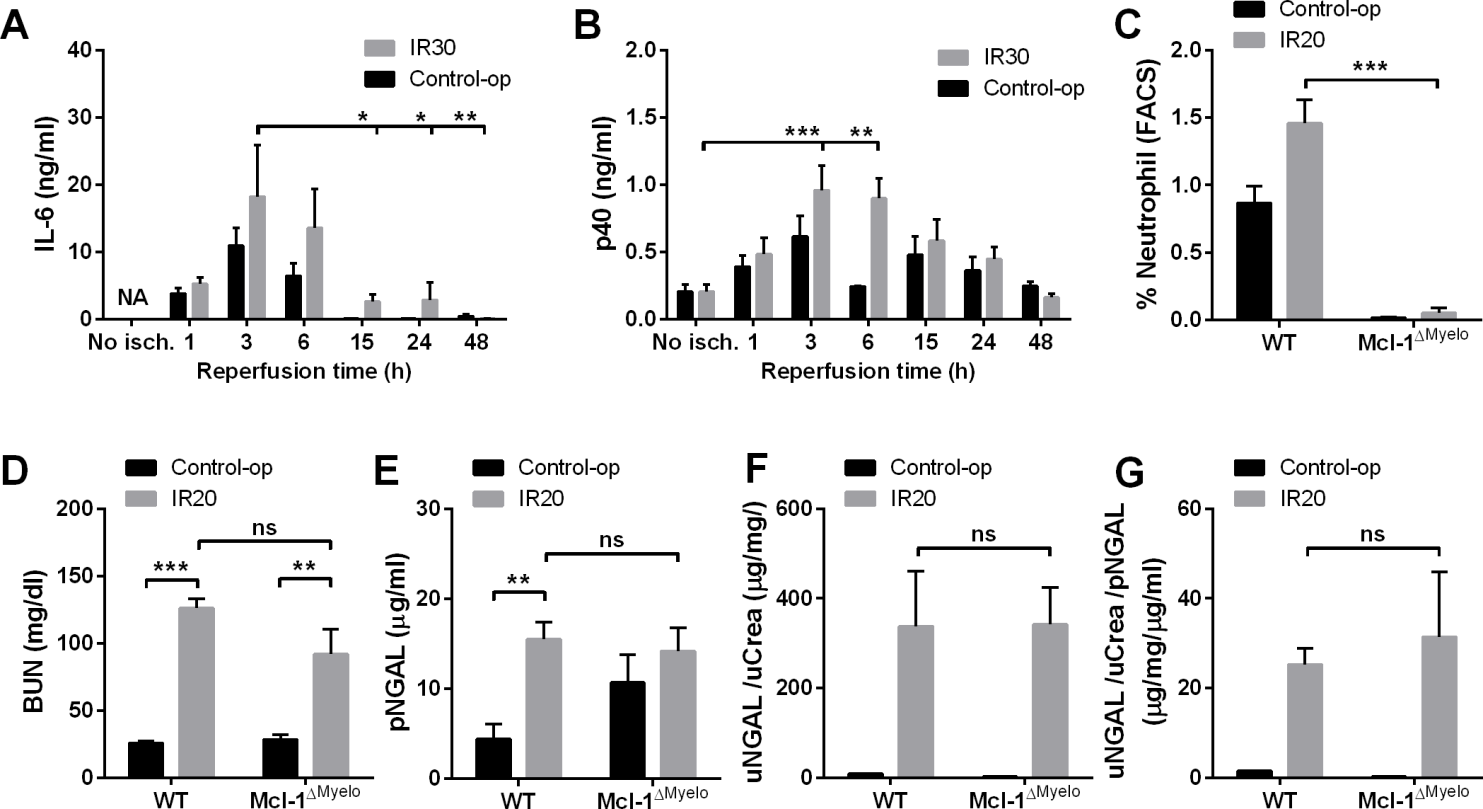
Inflammation after surgery induced NGAL production, but NGAL was not derived from neutrophils. Systemic inflammation markers (A) IL-6 and the (B) p40 subunit of IL-12/-23 peaked at 3 and 6 hours after both 30-min renal I/R injury and control. (C) In the Mcl-1^ΔMyelo^ bone marrow chimeric mice neutrophil deficiency was verified by FACS. There was no difference in (D) BUN, (E) pNGAL and (F, G) urinary NGAL levels normalized to urinary creatinine and to plasma NGAL in Mcl-1^ΔMyelo^ compared to wild type (WT) bone marrow chimeras. *, **, ***: p<0.05, 0.01, 0.001 vs. the groups indicated. (IL-6 and p40 kinetics n = 5/group/time-point; WT-control-op n = 7; WT-IR20 n = 17; Mcl-1^ΔMyelo^-control-op n = 6; Mcl-1^ΔMyelo^-IR20 n = 8).

To investigate the role of inflammation in NGAL induction IL-6 and IL-12/IL-23 total p40 plasma levels were monitored after renal ischemia and control operation. IL-6 levels rose similarly from undetectable levels to peak at 3 hours post-surgery both after 30-min ischemia and control operation (Fig 4A). The time-course of p40 plasma levels were similar to that of IL-6 without any difference between the control-operated and ischemic groups (Fig 4B).

The involvement of neutrophil granulocytes in NGAL production was also evaluated in Mcl-1^ΔMyelo^ chimeric mice with a myeloid-specific deletion of the anti-apoptotic myeloid cell leukemia-1 (MCL-1) factor resulting in neutrophil deficiency. In the absence of MCL-1 mice have a severe defect in neutrophil survival [32, 20]. Although a dramatic drop in neutrophil number was detected (Fig 4C) no decreases in plasma and urinary NGAL were revealed after severe (20-min) ischemia in Mcl-1^ΔMyelo^ compared to wild-type (WT) bone marrow chimeras (Fig 4D–4G).

All raw data are available in S1 Dataset.

## Discussion

Our results demonstrate that urinary NGAL (uNGAL) is a sensitive and specific marker of subclinical (10-min) renal ischemia, which is undetectable by blood urea nitrogen (BUN) in mice. NGAL is similarly sensitive to BUN in detecting severe kidney injury. Normalization of urinary NGAL to plasma NGAL (u/pNGAL) discriminates renal from non-renal injury. Finally, neutrophil deficiency did not alter NGAL upregulation after renal ischemia-reperfusion injury in mice, similarly to earlier findings [6], thus neutrophils are not a major source of NGAL in this model.

Compared to non-operated mice, renal NGAL mRNA (NGAL-mRNA) expression, and plasma and urinary NGAL (pNGAL and uNGAL) protein levels were elevated already by 10-min ischemia and were further elevated significantly from 10-min to more severe 20- or 30-min renal ischemia. There is growing evidence that plasma or urinary NGAL detects kidney injury before kidney function impairment in clinical trials [33, 34]. Hence, for the diagnosis of subclinical AKI, kidney damage markers, such as NGAL should be considered besides kidney function markers [35]. However, further studies are needed to introduce NGAL into the standard clinical practice [34, 36].

A baseline systemic NGAL production has been demonstrated previously [28, 37]. NGAL is filtered by the glomeruli [28], and is reabsorbed [18] and degraded [27] in proximal tubular cells [38]. However, the capacity of proximal tubular NGAL reabsorption could be saturated at relatively low plasma concentrations since in our study the intensity of renal cortical NGAL immunostaining was similar in all groups subjected to surgery. Low NGAL mRNA staining (with in situ hybridization) in spite of intense NGAL protein staining could demonstrate if this hypothesis is true or not. Punctate NGAL staining pattern in the renal cortex was present exclusively in the proximal tubules, therefore, they were probably endocytotic vacuoles suggesting NGAL reabsorption. NGAL staining increased dose-dependently after ischemia-reperfusion injury, as also reported previously [17].

After renal ischemia-reperfusion NGAL is mainly produced by the distal tubule [6]. In our study, renal NGAL production was evidenced by NGAL mRNA (NGAL-mRNA) in the kidney after ischemia-reperfusion. NGAL produced in the kidney is mainly excreted into the urine and enters the circulation to a lesser degree [19]. These previous findings suggest that urinary NGAL excretion increases much more in the case of kidney injury than due to injury of other organs. In fact, our study demonstrated that mild renal ischemia-reperfusion injury increased urinary NGAL (uNGAL) more than control operation, while plasma NGAL (pNGAL) was similar in the mild ischemia and control groups.

An intriguing finding was that plasma NGAL (pNGAL) concentration and renal NGAL mRNA (NGAL-mRNA) expression were similarly induced after control operation and 10-min ischemia. These results highlight the fact that the invasive surgery performed in control-operated mice led to significant inflammation. Inflammatory cytokines (IL-6 and p40 (subunit of both IL-12 and IL-23)) were elevated in the plasma demonstrating a prompt systemic inflammatory response that could contribute to the rise of plasma NGAL. Thus, systemic inflammation as a consequence of the operative procedure could induce extra-renal NGAL production, leading to filtration and tubular reabsorption of NGAL. However, the extent of inflammation was similar after control operation or even after severe (30-min) ischemia. On the other hand RAS activation induced by general anaesthesia [39, 40], and fluid loss during surgery may also lead to hypotension [41] and renal hypoperfusion, as we demonstrated renal NGAL mRNA elevation in control operated mice. We were able to observe a step-by-step elevation of uNGAL: first uNGAL increased in the control operation group compared to non-operated mice. Thereafter uNGAL gradually increased with the severity of ischemia, demonstrating a dose-response effect. Thus, we speculate that even a mild renal injury induced tubular secretion and/or reduced tubular reabsorption of NGAL. This theory is also supported by others [18].

Normalization of urinary NGAL to plasma NGAL (u/pNGAL) can possibly differentiate direct renal injury from extrarenal injury. Plasma to urinary NGAL (p/uNGAL) ratio has been proposed to distinguish septic from non-septic AKI in humans previously [42]. Moreover, an increase in the urinary to plasma NGAL ratio (u/pNGAL) but not plasma NGAL (pNGAL) was a good AKI marker in diabetic patients [43], and urinary to plasma NGAL ratio (u/pNGAL) was a slightly better AKI marker than urinary NGAL (uNGAL) alone. Our ROC curve analysis demonstrated that plasma or urinary NGAL (pNGAL or uNGAL) and urea (BUN) were similarly good markers of severe renal injury. Urinary NGAL (uNGAL) excretion and urinary to plasma NGAL ratio (u/pNGAL) were similarly useful to differentiate 10- and 20-min ischemia from control operation. However, urinary NGAL (uNGAL) was superior to plasma NGAL (pNGAL) to differentiate mild renal ischemia-reperfusion injury from control operation. The urinary to plasma NGAL ratio (u/pNGAL) did not show kidney injury in control-operated vs. non-operated mice, while it did show kidney injury after 10-min ischemia. These results support that urinary NGAL excretion normalized to plasma NGAL (u/pNGAL) is specific to ischemic kidney injury. The estimated fractional excretion of NGAL also supported the advantage of studying the ratio between urinary and plasma NGAL. Therefore, renal origin of urinary NGAL increase in pathologies leading not only to renal, but to other epithelial cell injury could be detected with the fractional excretion of NGAL. However we could calculate fractional excretion of NGAL in those groups only in which filtration markers did not change. Further studies would be needed to test fractional excretion of NGAL in more severe kidney injury that reduces GFR as well.

In cases when 24-hour urine collection is not feasible, concentration of excreted proteins such as NGAL (uNGAL) can be measured from spot urine and normalized to urine creatinine [44, 45, 46]. While the 24-hour urine normalization offers information about the average excretion in 24 hours, normalization to creatinine allows monitoring of the actual state of tubular injury. Here we demonstrated a significant correlation between the 24-hour and the creatinine normalized NGAL excretion suggesting that both spot urine and 24-hour urine are suitable to monitor tubular injury.

Limitations: in this study, the kidney function was measured only with blood urea nitrogen (BUN). Even though serum creatinine is a more commonly used kidney function marker in clinical practice, its levels were frequently unmeasurable in mice due to the low sensitivity of the available measuring method. However BUN increases similarly to creatinine in AKI mouse models [47, 48], therefore we used BUN.

In **conclusion** our study supports the diagnostic importance of NGAL in renal ischemia-reperfusion injury and provides comprehensive data about NGAL in this mouse model of AKI. Serious renal ischemia-reperfusion injury can be detected by conventional markers such as blood urea nitrogen retention with similar sensitivity to urinary or plasma NGAL. On the other hand, mild or very early stages of renal ischemia-reperfusion injury are only detected by urinary NGAL even before overall renal function starts to decline. Precaution is needed by the interpretation of plasma NGAL and if possible, the more sensitive and the more renal-specific urinary NGAL should be used to diagnose renal ischemia-reperfusion injury. Moreover, normalization of urinary NGAL to plasma NGAL could help to differentiate direct renal injury from extrarenal causes of NGAL elevation, especially if experimental circumstances require a sensitive distinction.

## Supporting Information

S1 Dataset Raw data for figures. (XLS)
